# Supplementary material for: WordCommentsAnalyzer: A windows software tool for qualitative research
Source: F1000Res. 2018 Sep 4;7:536. Originally published 2018 May 3. [Version 2] doi: 10.12688/f1000research.14819.2 (PMC6137411; doi:10.12688/f1000research.14819.2)
Supplement: Supplementary file 3 [file f1000research-7-17614-s0002.tgz › b2a79496-4ada-47c4-9682-a99ce867836d.zip!/Eight Iranian Health Journals/Archives of Rehabilitation_22.docx]

© Archives of Rehabilitation. This work is licensed under CC BY-NC 4.0 (https://creativecommons.org/licenses/by-nc/4.0/).

Volume & Issue: دوره 14 - شماره 2

Title: اثر تمرینات ثباتی بر پیامدهای عینی در بیماران مبتلا به کمردرد مزمن غیراختصاصی؛ مروری نظام‌مند بر مطالعات گذشته، با تأکید بر کارآزمایی‌های بالینی تصادفی

http://rehabilitationj.uswr.ac.ir/article-1-1272-fa.html

هدف: کمردرد از مشکلات شایع و پرهزینۀ بهداشتی‌درمانی است. تغییر الگوی حرکتی و نحوۀ به‌کارگیری عضلات عمقی در ناحیۀ کمر که مسئول حفظ ثبات ستون فقرات ناحیۀ کمری هستند، در افراد مبتلا به کمردرد گزارش شده است. برنامه‌های تمرینی مختلفی با هدف بهبود ثبات ستون فقرات ناحیۀ کمر، در درمان این بیماران استفاده می‌‌‌شود. هدف از این مطالعه، مرور نظام‌مند اثر تمرینات ثباتی بر پیامدهای عینی، در بیماران مبتلا به کمردرد مزمن غیراختصاصی، در مطالعات بالینی تصادفی گذشته است. روش بررسی: جستوجوی مطالعات انجام‌شده در فاصلۀ زمانی بین سال‌های ۲۰۱۲-۲۰۰۰ در منابع اطلاعاتی Google Scholar, PubMed, ScienceDirect, OVID, CINAHL and MEDLINE انجام شد. برای جست‌جو، از کلیدواژه‌هایی مانند کمردرد مزمن، تمرینات ثباتی، عضلات ستون فقرات، عضلۀ ترنسورس ابدومینیس، عضلۀ مولتی فیدوس، الکترومیوگرافی، اولتراسونوگرافی و مطالعۀ تجربی تصادفی استفاده شده است. پیامدهای عینی که در این مطالعه بررسی شده است، الگوی فعالیت عضلانی، اندازۀ عضلات، کنترل تعادل پوسچرال، استقامت عضلات و دامنۀ حرکتی ناحیۀ کمر بوده است. یافته‌ها: درکل، از بین ۱۵۸ مطالعه، ۱۳ مقاله‌ای انتخاب شدند که معیارهای ورود را داشتند. تفاوت‌های بسیاری میان مقالات، ازنظر متدولوژی، شاخص‌های اصلی ارزیابی‌شده، تعداد نمونه، روش اجرا، شاخص و... مشاهده شد. یافته‌ها نشان می‌‌‌دهد که تمرینات ثباتی، درمقایسه با سایر مداخلات درمانی، بر بهبود فعالیت خودکار خودکار عضلات عمقی و کنترل تعادل و استقامت عضلات تنه اثر معنی‌داری داشته است؛ درحالی که درزمینۀ تأثیر بر اندازۀ عضله، تفاوت معنی‌داری با سایر مداخلات درمانی نداشته است. نتیجه‌گیری: به‌نظر می‌‌‌رسد که تمرینات ثباتی اثر درخور توجهی در بهبود الگوی فعالیت عضلانی، تعادل پوسچرال، استقامت عضلانی و دامنۀ حرکتی افراد مبتلا به کمردرد داشته باشد. همچنین یکی از مداخلات مؤثر درزمینۀ درمان بیماران مبتلا به کمردرد محسوب می‌‌‌شود.

Title: تأثیر برنامۀ بازورود به جامعه، بر وضعیت روانی بیماران اسکیزوفرنیا

http://rehabilitationj.uswr.ac.ir/article-1-1197-fa.html

هدف: پژوهش حاضر با هدف تعیین تأثیر «برنامۀ بازورود به جامعه» بر وضعیت روانی بیماران مبتلا به اسکیزوفرنیا انجام شده است. روش بررسی: در این مطالعۀ نیمه‌تجربی که نمونه‌گیری از جامعه به‌صورت در دسترس و غیراحتمالی انجام شد، ۲۴ بیمار مبتلا به اسکیزوفرنیا انتخاب شدند که معیارهای ورود به مطالعه را داشتند و در دو گروه مداخله (۱۲ نفر) و کنترل (۱۲ نفر) قرار گرفتند. اطلاعات با استفاده از پرسش‌نامۀ «مقیاس مجموعه علائم مثبت و منفی» و با روش مصاحبه جمع‌آوری شد. گروه مداخله به‌صورت عملی و نظری «برنامۀ بازورود به جامعه» را آموزش دیدند. برنامه شامل ۱۶ جلسۀ آموزشی بود که به‌صورت یک روز در میان و ۳ بار در هفته اجرا شد. بیماران گروه کنترل در طولِ اجرای برنامه فقط از درمان‌های معمول بیماران بستری در بیمارستان‌های روان‌پزشکی، شامل دارودرمانی و کاردرمانی، استفاده می‌کردند. داده‌های به‌دست‌آمده از پژوهش با استفاده از آزمون تی برای دو گروه مستقل تحلیل شد. یافته‌ها: یافته‌ها بیانگر این است که علائم منفی بیماران اسکیزوفرنیای گروه مداخله در‌مقایسه‌با گروه کنترل، پس از اجرای برنامۀ بازورود به جامعه، تغییر معناداری نشان می‌دهد (۰/۰۵>P). هرچند تغییر در میانگین کل و ابعاد دیگر آن مشهود بود، ازنظر آماری تغییرات مربوط به این ابعاد شامل علائم مثبت و وضعیت روان‌شناسی عمومی و نمرۀ کل، معنی‌دار نیست (P<0/05). نتیجه‌گیری: براساس یافته‌های پژوهش، برنامۀ بازورود به جامعه باعث مهار و بهبود علائم منفی بیماران اسکیزوفرنیا می‌شود که می‌توان این تفاوت معنی‌دار را در گروه مداخله به تأثیرگذاربودن برنامه نسبت داد؛ در‌حالی‌که در گروه کنترل این تفاوت مشاهده نشد.

Title: لب‌خوانی و ادراک گفتار دانش‌آموزان کم‌شنوای مدارس ویژۀ کم‌شنوایان در شهر تهران

http://rehabilitationj.uswr.ac.ir/article-1-1122-fa.html

هدف: هدف از پژوهش حاضر، بررسی‌ توانایی لب‌خوانی و ادراک گفتار دانش‌آموزان کم‌شنوای مدارس ویژۀ کم‌شنوایان در شهر تهران و در سطوح مختلف گفتاری بود. روش بررسی: در این مطالعۀ توصیفی‌تحلیلی، ۴۴ دانش‌آموز کم‌شنوا (۹ تا ۱۲ساله)، از دو مدرسۀ ویژۀ کم‌شنوایان شهر تهران (دخترانه و پسرانه)، با استفاده از روش نمونه‌گیری خوشه‌ای و چند‌مرحله‌ای انتخاب شدند. ابزارهای این پژوهش، پرسش‌نامۀ محقق‌ساخته است که برای سنجش اطلاعات دموگرافیک نمونه‌ها، آزمون ادراک شنوایی APT، آزمون لب‌خوانی شمارۀ ۲ سارا به دو شیوۀ بدون ‌صدا و باصدا و آزمون تمایز گفتار SDS به دوشیوۀ بدون ‌صدا و باصدا استفاده می‌شود. برای تجزیه‌وتحلیل یافته‌ها از آزمون‌‌های تی وابسته و مستقل استفاده شد.  یافته‌ها: لب‌خوانی و تمایز گفتار ۹۷٫۳ درصد دانش‌آموزان، در وضعیت بسیار ضعیفی قرار داشت و تنها ۶۷ درصد از آنان، ادراک شنوایی مسلطی داشتند (۰/۰۰۱>P). بین امتیازات آزمودنی‌ها در دو حالت بدون ‌صدا و باصدای آزمون سارا (واج‌ها)، تفاوت معناداری وجود داشت (۰/۰۰۱>P)؛ اما بین امتیازات آزمودنیها در حالت بدون ‌صدا و باصدای آزمون SDS (واژه‌ها) تفاوت معناداری وجود نداشت. همچنین، در هیچ‌یک از آزمون‌‌های پژوهش، تفاوت معناداری بین دختران و پسران مشاهده نشد. نتیجه‌گیری: لب‌خوانی و ادراک گفتار، اولین شاخص روش آموزش شفاهی است. این شاخص در دانش‌آموزان کم‌شنوای مدارس ویژۀ کم‌شنوایان در شهر تهران، وضعیت مناسبی نداشت. برای بررسی راه‌حل‌‌های مناسب، پژوهش‌‌‌های بیشتری لازم است.

Title: تأثیر تمرین قدرتی و برنامه بازتوانی قلبی بر پارامترهای بیومکانیکی سرعت و شدت جریان خون و ارتباط آن با سختی رگ در عروق بازویی و رانی در بیماران پیوند عروق کرونری

http://rehabilitationj.uswr.ac.ir/article-1-1121-fa.html

هدف: با فرض اینکه بازتوانی قلبی و ورزش سبب کاهش قابل توجهی در میزان مرگ و میر بیماران عروق کرونر قلب می‎شود و نقش مهمی‌ در امر پیشگیری ثانویه دارد، هدف از انجام این تحقیق اثر تمرینات قدرتی و برنامه بازتوانی قلبی بر پارامتر‎های بیومکانیکی سرعت و شدت جریان خون و بررسی ارتباط آن با شاخص سختی رگ در ‎شریان‌های بازویی و رانی بیمارانی است که یک تا دو ماه از عمل پیوند عروق کرونری آنها گذشته است. روش بررسی: این پژوهش به صورت پیش‎آزمون و پس‎آزمون روی ۴۰ زن و مرد ۵۰ تا ۸۰ سالی که تحت عمل بای‎پس عروق کرونری قرار گرفته بودند، انجام شد. آزمودنی‎های گروه آزمایش را ۱۸ نفر که به مدت شش هفته، هفته‎ایی سه جلسه به انجام برنامه بازتوانی و تمرینات قدرتی با شدت‎ معین انجام شد، تشکیل دادند. گروه کنترل ۱۴ نفر بود، که در برنامه‎ی تمرینی شرکت نداشتند. اندازه‎گیری متغیرهای سرعت جریان خون و قطر رگ در فاز سیستول و دیاستول با دستگاه اولتراسوند داپلر اندازه‎گیری و میانگین سرعت، شدت جریان خون و شاخص سختی‎رگ برای هر دو گروه محاسبه شد. از تی تست وابسته در سطح معناداری ۰/۰۵ از ضریب همبستگی پیرسون جهت ارتباط سنجی بین پارامتر‎ها در سطح معناداری ۰٫۰۱ جهت تحلیل آماری استفاده شد.  یافته‌ها: تغییرات معنا‎داری در میزان اختلاف میانگین قطر سیستول و دیاستول شریان رانی و بازویی (۰/۰۰=P)، سرعت جریان سیستول شریان بازویی و رانی (۰/۰۴ و ۰/۰۰=P) و دیاستول آن(۰٫۰۰=P)، فشار خون سیستول و دیاستول (۰/۰۱ و ۰/۰۰=P) در گروه آزمایش وجود داشت. شاخص سختی ‎رگ و شدت جریان خون بیماران گروه آزمایش در ‎شریان‌ها تغییر معناداری نداشت. نتیجه‌گیری: با توجه به نتایج بدست‎آمده، می‎توان گفت تمرینات قدرتی و برنامه بازتوانی قلبی می‎تواند در بهبود رفتار همورئولوژی خون و وضعیت عروق محیطی این بیماران موثر باشد.

Title: ارزیابی سطح مهارت‌های تکامل فردی‌-اجتماعی کودکان مبتلا به فنیل‌کتونوری تحت رژیم‌درمانی از بدو تولد

http://rehabilitationj.uswr.ac.ir/article-1-1163-fa.html

هدف: این مطالعه با هدف تعیین سطح مهارت‌های تکامل فردی‌اجتماعی کودکان ۴-۱ ساله‌ای طرح‌ریزی شده است که از بدو تولد به فنیل‌کتونوری تحت رژیم‌درمانی مبتلا بوده‌اند. روش بررسی: این مطالعه به روش مقطعی‌تحلیلی دربارۀ ۴۰ کودک ۱ تا ۴ساله انجام شد. انتخاب این کودکان، براساس فهرست بیماران کلینیک فنیل‌کتونوری بود که از بدو تولد، غربالگری شده و تحت‌ درمان قرار گرفته بودند. ابزار گردآوری اطلاعات، پرسش‌نامۀ دموگرافیک و پرسش‌نامۀ ASQ و ارزیابی بالینی توسط متخصص کودکان بود. ۴۰ کودک نیز به‌عنوان گروه کنترل، به‌طور تصادفی از مهدکودک‌های تهران انتخاب شده و پرسش‌نامه‌های یادشده برای آن‌ها تکمیل شد. تحلیل داده‌ها نیز با استفاده از نرم‌افزار SPSS، نسخۀ ۱۹ انجام شد. یافته‌ها: متوسط سن کودکان ۳۰/۷ (± ۱۰/۵) ماه در گروه کنترل و ۲۷/۷ (± ۱۱/۰) ماه در گروه مورد بود. بین دو گروه، ازنظر نحوۀ زایمان، دور سر، قد و وزن فعلی و وزن بدو تولد رابطۀ معناداری وجود نداشت؛ اما ازنظر تکامل مهارت‌های فردی‌اجتماعی، اختلاف معناداری وجود داشت (۰/۰۰۱>P). نتیجه‌گیری: براساس نتایج این مطالعه، سطح تکامل فردی‌اجتماعی در کودکان درمان‌شده با رژیم‌درمانی از بدو تولد، کمتر از کودکان عادی است؛ لذا تشخیص و مداخلۀ زودرس برای همۀ کودکان درمان‌شده با رژیم‌درمانی، از بدو تولد و در سنین کم توصیه می‌شود.

Title: تأثیر هشت هفته فعالیت ورزشی هوازی بر برخی عوامل قلبی‌عروقی و تنفسی و لیپیدی جانبازان مصدوم نخاعی

http://rehabilitationj.uswr.ac.ir/article-1-1102-fa.html

هدف: مصدومان نخاعی به‌علت بی‌تحرکی، ازنظر بیماری‌های قلبی‌تنفسی، در معرض خطر بیشتر قرار دارند. هدف مطالعۀ حاضر، بررسی تأثیر ۸ هفته فعالیت ورزشیِ هوازی بر برخی عوامل قلبی‌عروقی و تنفسی و لیپیدی جانبازان مصدوم نخاعی بود. روش بررسی: ۲۰ نفر از جانبازان مصدوم نخاعی بالای ۷۰ درصد شهر اصفهان با سن ۴/۵ ± ۴۹/۹ سال و سابقۀ ابتلای ۱/۸ ± ۲۵/۹ سال و نمایۀ تودۀ بدن ۳/۹ ± ۲۵/۰۳ کیلوگرم‌برمترمربع به‌طور تصادفی، به دو گروه تجربی و کنترل تقسیم شدند. قبل و بعد از مداخله (۳۰ دقیقه فعالیت ورزشی هوازی به‌مدت ۸ هفته و ۳ جلسه)، فشارخون سیستولی و دیاستولی، ظرفیت حیاتی VC، ظرفیت بازدمی اجباری یک ثانیه (۱FEV)، HDL-C، LDL-C و قندخون ناشتای دو گروه تجربی و کنترل اندازه‌گیری شدند. داده‌ها با استفاده از آزمون آماری اندازه‌گیری‌های مکرر با سطح معنی‌داری ۰/۰۵>P تجزیه‌وتحلیل شد. یافته‌ها: فشارخون سیستولی (LDL–C) (P=0/004) و (P=0/002) گروه تجربی پس از فعالیت ورزشی کاهش معنی‌داری پیدا کرد. ظرفیت حیاتی P=0/01) و (P=0/031 و (  (P=0/004پس از مداخله، افزایش معنی‌داری داشته است. پس از مداخله در دو گروه تجربی و کنترل، تفاوت معنی‌داری بین این متغیرها مشاهده نشده است .(P>0/05) نتیجه‌گیری: باتوجه‌به داده‌های به‌دست‌آمده از مطالعه، به‌نظر می‌رسد یک دوره فعالیت ورزشی (۸ هفته) موجب بهبود وضعیت تنفسی و قلبی‌عروقی و لیپیدی جانبازان مصدوم نخاعی می‌شود.

Title: تأثیر آموزش نظریۀ ذهن بر بهبود مهارت‌های اجتماعی دانش‌آموزان با ناتوانی هوشی تحولی

http://rehabilitationj.uswr.ac.ir/article-1-1081-fa.html

هدف: تحول نظریۀ ذهن در کودکان، به‌عنوان یکی از جنبه‌های شناخت اجتماعی، در سال‌های اخیر پژوهشگران متعددی را به خود متوجه کرده است. هدف پژوهش، تعیین تأثیر آموزش نظریۀ ذهن بر مهارت‌های اجتماعی دانش‌آموزان پسر دچار ناتوانی هوشی تحولی در مشکین‌شهر بود. روش بررسی: در مطالعۀ آزمایشی حاضر، از طرح پیش‌آزمون‌ ـ پس‌آزمون با گروه کنترل استفاده شد. دانش‌آموزان پسر دچار ناتوانی هوشی تحولی ۸تا۱۲ سال شاغل به تحصیل در مشکین‌شهر (۴۳ نفر) به روش تمام‌شماری، به آزمون‌های نظریۀ ذهن پاسخ دادند. دانش‌آموزانی که در گذراندن آزمون‌ها موفق نشدند (۳۹ نفر) به‌عنوان نمونه انتخاب شدند و معلم‌های آن‌ها مقیاس درجه‌بندی مهارت‌های اجتماعی (گرشام و الیوت، ۱۹۹۰) را تکمیل کردند. سپس دانش‌آموزان به دو گروه کنترل و آزمایش تقسیم شدند. گروه آزمایش در ۸ جلسۀ آموزشی (به‌مدت ۲ هفته، هر جلسه ۳۰ دقیقه) شرکت کردند. بعد از پایان جلسات آموزشی، تمامی‌دانش‌آموزان با آزمون‌های نظریۀ ذهن و مقیاس درجه‌بندی مهارت‌های اجتماعی مجدداً ارزیابی شدند. داده‌ها با استفاده از تحلیل هم‌پراکنش تحلیل شد.  یافته‌ها: نتایج تحلیل هم‌پراکنش نشان داد که پس از مداخلۀ آموزشی، عملکرد گروه آزمایش در شاخص مهارت‌های اجتماعی و همکاری و خویشتن‌داری، به‌طور معنا‌داری (۰/۰۰۱=P) بالاتر از گروه کنترل بود؛ اگرچه، دو گروه در مؤلفۀ ابراز وجود با یکدیگر تفاوت معنادار نداشتند.  نتیجه‌گیری: آموزش نظریۀ ذهن به دانش‌آموزان دچار ناتوانی هوشی تحولی، موجب بهبود مهارت‌های اجتماعی و مؤلفه‌های آن می‌شود و ضامن موفقیت آن‌ها در تعامل‌های اجتماعی در بزرگ‌سالی به‌شمار می‌آید.

Title: یادگیری حرکتی سکانسی صریح بیماران مبتلا به ام اس از نوع Relapse-Remission

http://rehabilitationj.uswr.ac.ir/article-1-1209-fa.html

هدف: بررسی یادگیری حرکتی صریح در بیماران مبتلا به MS از نوعِ Relapse-remission و مقایسۀ آن با افراد سالم، هدف این تحقیق است. روش بررسی: برای بررسی یادگیری حرکتی صریح، از وظیفه‌ای حرکتی استفاده شد که با استفاده از نرم‌افزار اجرا می‌شد. ۱۵بیمار مبتلا به RRMS و ۱۵فرد سالم که با گروه بیمار همسان شده بودند، در این تحقیق شرکت کردند. وظیفۀ حرکتی بدین‌صورت بود که ۴ مربع با رنگ‌‌های مختلف روی صفحۀ نمایش رایانه ظاهر می‌شد و داوطلب می‌بایست به‌محض مشاهدۀ هر مربع، کلید مربوط‌به آن را فشار می‌دادو ۴۸ ساعت بعد،از افراد آزمون یادآوری گرفته می‌شد.آزمون روز اول، شامل ۸ بلوک و آزمون یادآوری شامل ۲ بلوک منظم بود. قبل از آزمون، توضیحات لازم درخصوص الگوی بلوک‌ها به فرد داده می‌شد. زمان اجرای آزمایش، بلوک‌‌های تحقیق به‌عنوان متغیر وابسته اندازه‌گیری شده و با استفاده از آزمون‌‌های K-S وتی زوج‌شده وتیمستقل تحلیل شدند. یافته‌‌ها: انجام بلوک‌های تحقیق در بیماران به‌صورت معنی‌داری کندتر از افراد سالم بود (۰/۰۵>P). اختلاف زمانی بلوک دهم درمقایسه‌بابلوک اول در هر دو گروه معنی‌دار بود (۰/۰۵>P) و یادگیری حرکتی صریح در افراد سالم بیشتر از افراد بیمار بود (۰/۰۵>P). نتیجه‌گیری: اگرچه گروه بیمار وظیفۀ حرکتی را کندتر از گروه سالم انجام دادند، یادگیری حرکتی صریح در این گروه نیز وجود داشت؛اما میزان یادگیری حرکتی صریح دربیماران،درمقایسه‌با گروه سالمِ همسان خود کمتر بود.

Title: مقایسۀ دو رویکرد تانسیون‌ اکتیو زودهنگام و بی‌حرکتی در توان‌بخشی، پس از ترمیم جراحی نواحی ۵‌و‌۶ تاندون‌های اکستانسوری دست

http://rehabilitationj.uswr.ac.ir/article-1-1212-fa.html

هدف: هدف از پژوهش حاضر، بررسی و مقایسۀ دو روش «بی‌حرکتی» و «تانسیون‌ اکتیو زودهنگام» در توان‌بخشی پس از ترمیم جراحی آسیب تاندون‌های اکستنسوری در نواحی ۵‌ و‌ ۶ دست بود. روش بررسی: از ۲۱ بیمار واردشده به این مطالعۀ نیمه‌تجربی و از نوع کارآزماییِ بالینیِ تصادفی، ۱۸ بیمار با میانگین سنی ۴۳/۳۲ درمان را کامل کردند. درمجموع، ۴۱ تاندون اکستنسور بریده‌شده در ۲۷ انگشت این افراد مطالعه شد. ترمیم همۀ تاندون‌ها در ناحیۀ ۶ و ۵ با استفاده از تکنیک جراحی رانینگ انتهابه‌انتهای اپی‌تنون و با پرولن ۰/۳ را یک جراح انجام داد. برای توانبخشی، افراد به‌صورت تصادفی در یکی از دو گروه درمان مرسوم، با «روش بی‌حرکتی» و «تانسیون اکتیو زودهنگام» قرار گرفتند. در هفتۀ ششم بعد از عمل جراحی، میزان اِدم پس از جراحی دست، فلکسیون در مفصل متاکارپوفالانژیال، فلکسیون و اکستانسیون ترکیبی مچ و انگشتان، حرکت اکتیو کلی انگشتان و طویل‌شدگی اکستنسوری اندازه‌گیری شد. در هفتۀ دوازدهم، علاوه‌بر تمام شاخص‌های بیان‌شده، قدرت گریپ نیز اندازه‌گیری شد. یافته‌ها: درمقایسه‌با روش مرسوم بی‌حرکتی، درمان به‌روش تانسیون اکتیو زودهنگام باعث بهبود معنی‌دار شاخص‌های اِدم پس از جراحی دست، فلکسیون در مفصل متاکارپوفالانژیال انگشتان، فلکسیون ترکیبی مچ و انگشت، حرکت اکتیو کلی انگشتان و قدرت گریپ دست شد (۰/۰۵>P). در شاخص‌های اکستانسیون ترکیبی مچ و انگشتان و طویل‌شدگی اکستنسوری بین دو گروه، تفاوت معنی‌داری یافت نشد (۰/۰۵>P). نتیجه‌گیری: باتوجه‌به یافته‌ها، روش تانسیون اکتیو زودهنگام رویکردی است که می‌توان آن را جایگزین مناسبی برای روش مرسوم بی‌حرکتی، در توانبخشی بعد از عمل ترمیم جراحی تاندون‌های اکستنسوری نواحی ۵و۶، برای بیماران سازگار درنظر گرفت.

Title: بررسی اثر روش درمانی dry needling ومقایسۀ آن باروش فشار ایسکمیک برشدت درد نقطۀ ماشه‌ای فعال در عضلۀ تراپز فوقانی

http://rehabilitationj.uswr.ac.ir/article-1-1176-fa.html

هدف: نقطۀ ماشه‌ای از علت‌های بسیار مهم ایجاد درد و اختلالات اسکلتی‌عضلانی است. وجود نقطۀ ماشه‌ای در عضلۀ تراپز فوقانی، یافته‌ای شایع در افراد مبتلابه دردهایناحیۀ گردن و پشت است.هدف از این مطالعه، بررسی اثر سوزن خشک در مقایسه‌ با فشار ایسکمیک بر نقطۀ ماشه‌ای فعال در عضلۀ تراپز فوقانی است. روش بررسی: در این مطالعۀکارآزمایی بالینی تصادفی،۳۲خانمرا انتخاب کردند که نقاط ماشه‌ای فعال در عضلۀ تراپز فوقانی دارند. افراد به‌طور تصادفی در دو گروه درمانی قرار گرفتند:۱. گروه تحت درمان سوزن خشک(۱۵نفر)؛ ۲. گروه تحت درمان فشار ایسکمیک (۱۷ نفر). از مقیاس دیداری درد، برای اندازه‌گیری شدت درد قبل و بعد از درمان، در هر دو گروه استفاده شد. به‌منظور بررسی تغییرات شدت درد پس از درمان، درمقایسه‌با مقادیر قبل از درمان، در هر دو گروه درمانیاز آزمونpaired t-test استفاده شد. برای تعیین وجود اختلاف بین دو روش درمانی از آزمون تحلیل هم‌پراکنش استفاده شد. یافته‌ها: تجزیه‌وتحلیل آماری،کاهش معنی‌دار شدت درد را بعد از درمان، درمقایسه‌با قبل از درمان، در هر دو گروه درمانی نشان داد (۰/۰۰=P). در آزمون تحلیل هم‌پراکنش تفاوت معنی‌داریبین دو گروه یافت نشد (۰/۸=P). نتیجه‌گیری: به‌نظر می‌رسد،در بهبود شدت درد افرادی کهنقاط ماشه‌ای فعال در عضلۀ تراپز فوقانی دارند، هر دو روش درمانی مؤثر است. بنابراین درمانگران و فیزیوتراپیست‌ها می‌توانند روش درمانی سوزن خشکرا نیزدر کلینیک‌های فیزیوتراپی استفاده کنند.

Title: بررسی سطح استرگنوزیس دست در کودکان مبتلا به دای‌پلژی و همی‌پلژی اسپاستیک ۳ تا ۶سال

http://rehabilitationj.uswr.ac.ir/article-1-864-fa.html

هدف: یکی از مشکلات حسی شایع در فلج مغزی،آسترگنوزیس است که اهمیت ویژه‌ای در عملکردهای دستی روزمره دارد. هدف از مطالعۀ حاضر، بررسی سطح استرگنوزیس دست درکودکان مبتلا به دای‌پلژی اسپاستیک و همی‌پلژی اسپاستیک بود. روش بررسی: در این مطالعۀ مقطعی، ۲۰ کودک ۶-۳ سال مبتلا به فلج مغزی (۹ پسر و ۱۱ دختر)بامیانگین سنی «همی‌پلژی: ۵۵ ماه، دای‌پلژی: ۵۷ ماه» به روش غیراحتمالی ساده، از میان مراجعه‌‌کنندگان به مراکز کاردرمانی دانشگاه علوم پزشکی شهیدبهشتی انتخاب شدند. استرگنوزیس با استفاده از اشکال هندسی (مربع، دایره، مثلث و مستطیل) و اشیای معمولی (مداد، کلید، سکه، میخ، قاشق چای‌خوری و پیچ) و تختۀ مخصوص تست بررسی شد. اطلاعات با استفاده از آزمون‌های تحلیل پراکنش مخلوط و رگرسیون، تجزیه‌ و‌ تحلیل گردید. یافته‌ها: ارتباط معناداری بین سن و نمرۀاسترگنوزیس اشیای معمولی در کودکان مبتلا به همی‌پلژی مشاهده نشد؛ ولی ارتباط بین سن و نمرۀ استرگنوزیس اشکال هندسی در این کودکان معنادار بود (۰/۰۲۷=P). بین سن و نمرۀاسترگنوزیس اشیای معمولی و اشکال هندسی در کودکان مبتلا به دای‌پلژی نیز ارتباط معناداری مشاهده نشد. اثر اصلی جنس در استرگنوزیس کودکان مبتلا به همی‌پلژی و دای‌پلژی معنادار نبود. اثر اصلی دست نیز در استرگنوزیس هیچ‌یک از دو گروه معنادار نبود. نتیجه‌گیری: استرگنوزیس دست سالم و مبتلا در کودکان مبتلا به همی‌پلژی و دست راست و چپ کودکان مبتلا به دای‌پلژی، تفاوت معنا‌داری نداشت. همچنین، ارتباط معناداری بین سن و نمرۀ استرگنوزیس اشکال هندسی در کودکان مبتلا به همی‌پلژی وجود داشت.

Title: بررسی رابطۀپرخاشگری و تکانشگری با آمادگی به اعتیاد، در دانشجویان پسر

http://rehabilitationj.uswr.ac.ir/article-1-1220-fa.html

هدف: پژوهش حاضر،با هدف بررسی رابطۀ پرخاشگری و تکانشگری با آمادگی به اعتیاد در دانشجویان دانشگاه علامه‌طباطبایی انجام شده است. روش بررسی: روش استفاده‌شده در این پژوهش،از نوع مقطعی‌تحلیلی بود. دراین مطالعه،۱۷۰ نفرازدانشجویان پسر در مقطع کارشناسی دانشگاه علامه‌طباطبایی که با روش نمونه‌گیری دومرحله‌ای‌ تصادفی انتخاب شده بودند، تحت‌مطالعه قرار گرفتند. برای سنجش متغیرها، از پرسش‌نامۀ پرخاشگری اهواز، مقیاس تکانشگری بارات (BIS) و مقیاس آمادگی به اعتیاد وید و بوچر استفاده شد. برای تحلیل یافته‌ها از ضریب هم‌بستگی پیرسون و رگرسیون چندگانه استفاده شد. یافته‌ها: نتایج نشان داد که متغیرهای پرخاشگری و تکانشگری به‌طور معناداری توانایی پیش‌بینی آمادگی به اعتیاد را دارند (۰/۰۰۱>P). همچنین، برمبنای ضریب تعیین متغیرهای پرخاشگری و تکانشگری باهم ۴۹ درصد از پراکنش متغیر آمادگی به اعتیاد را پیش‌بینی کردند. نتیجه‌گیری: باتوجه‌به اینکه متغیرهای پرخاشگری و تکانشگری، اعتیادپذیری را در دانشجویان به‌خوبی پیش‌بینی می‌کنند، مراکز مشاورۀ دانشگاه‌ها می‌توانند با برگزاری کارگاه‌هایی، شیوه‌های مهار این متغیرها را در موقعیت‌های مختلف به دانشجویان آموزش دهند و ازاین‌طریق، از گرایش آن‌ها به‌مصرف مواد پیشگیری کنند.

Title: حمایت اجتماعی و اضطراب در سالمندان شهر همدان

http://rehabilitationj.uswr.ac.ir/article-1-1210-fa.html

هدف: امروزه باتوجه‌به میزان رشد درخور توجه سالمندی در ایران، بیش از گذشته به حفظ و ارتقای سلامت و کیفیت زندگی سالمندان به‌عنوان یک گروه جمعیتی آسیب‌پذیر توجه می‌شود. هدف از انجام‌دادن این مطالعه، بررسی رابطۀ حمایت اجتماعی و اضطراب در سالمندان است. روش بررسی: در این مطالعۀ مقطعی که به روش توصیفی‌تحلیلی انجام گرفته است، ۳۸۰ نفر از سالمندان شهر همدان با روش نمونه‌گیری دردسترس بررسی شدند. به‌منظور جمع‌آوری اطلاعات، علاوه‌بر پرسش‌نامۀ اطلاعات دموگرافیک، از پرسش‌نامۀ حمایت اجتماعی و مقیاس اضطراب سالمندان نیزاستفاده شد. برای تجزیه‌و‌تحلیل داده‌ها نیز از آزمون‌های آماری ضریب هم‌بستگی و تی مستقل استفاده شد. یافته‌ها: ۳۸۰ سالمند در مطالعه شرکت کردند که ۶۸/۹ درصد مرد و ۹۰ درصد متأهل بودند. متوسط سن سالمندان در این پژوهش، ۶۸/۰۶ سال با انحراف‌معیار ۶/۷۲ بود. براساس یافته‌های پژوهش،حمایت اجتماعی با اضطراب و تمامی‌خرده‌مقیاس‌های آن رابطۀ منفی و معناداری دارد که دراین‌بین، خرده‌مقیاس شناختی بیشترین هم‌بستگی را نشان می‌دهد (۰/۰۰۱>P و ۰/۴۹۴-=r). نتیجه‌گیری: باتوجه‌ به روند روبه‌رشد سالمندی، توجه به تعیین‌کنندۀ اجتماعی سلامت مانند «حمایت اجتماعی»،برای کاهش اضطراب سالمندان ضروری است و اهمیت خاصی دارد.

Title: بررسی روایی و پایایی نسخۀ فارسی پرسش‌نامۀIKDCبرای ارزیابی وضعیت بیماران ایرانی، پس از جراحی رباط صلیبی قدامی‌ و منیسک زانو

http://rehabilitationj.uswr.ac.ir/article-1-1214-fa.html

هدف: برای ارزیابی پیامدهای بیماران مبتلا به صدمات زانو، پایایی پرسش‌نامۀ International Knee Documentation Committee (IKDC)ازنظر بین‌المللی تأییدشده است. هدف تحقیق حاضر، ترجمه و بررسی تکرارپذیری در دفعات آزمون وهمخوانی درونی و پایایی سازۀ نسخۀ فارسی پرسش‌نامۀ IKDC در بیماران ایرانی، پس از جراحی رباط صلیبی قدامی‌ و منیسک زانوست. روش بررسی: نسخۀ فارسی پرسش‌نامه‌های IKDCوKOOS و ۳۶-SF، بعد از ترجمه، براساس روش ارزیابی بین‌المللی کیفیت زندگی، در ۱۰۱ بیمار، پس از جراحی رباط صلیبی قدامی‌ و منیسک زانو موردِاستفاده قرار گرفت. تکرارپذیری در دفعات آزمون، با شاخص آماری ICC و همخوانی درونی با استفاده از ضریب آلفای کرونباخ بررسی شد. پایایی سازه ازطریق بررسی ارتباط نمرۀ پرسش‌نامۀIKDCبا نمرۀ خرده‌مقیاس‌های پرسش‌نامۀKOOS و خلاصه سلامت جسمانی و روانی پرسش‌نامۀ۳۶-SFارزیابی شد. به‌علاوه برای بررسی پایایی، از ضرایب هم‌بستگی اسپیرمن و پیرسون استفاده گردید. یافته‌ها: تکرارپذیری نسخۀ ترجمه‌شده، ۰/۹۹ است که بسیار بالاست. همخوانی درونی نمرات به‌دست‌آمدۀ آیتم‌ها، ۹۰ درصد بود. هم‌بستگی آماری خوبی بین نمرۀ کل نسخۀ فارسی IKDC و خلاصه سلامت جسمانی پرسش‌نامۀ۳۶-SF (با ضریب هم‌بستگی ۷۰ درصد و سطح معنی‌داری کمتر از ۰/۰۰۱) وجود داشت؛ ولی بین نمرۀ کل و خلاصه سلامت روانی (ضریب هم‌بستگی ۰/۴۴) هم‌بستگی آماری کم بود. همچنین، هم‌بستگی آماری خوبی با خرده‌مقیاس درد در پرسش‌نامۀ KOOS (ضریب هم‌بستگی ۰/۶۶)، هم‌بستگی متوسطی با خرده‌مقیاس علائم بالینی (ضریب هم‌بستگی ۰/۵۵)، هم‌بستگی خوبی با خرده‌مقیاس فعالیت روزمره (ضریب هم‌بستگی ۰/۶۸)، هم‌بستگی متوسطی با خرده‌مقیاس فعالیت ورزشی و تفریحی (ضریب هم‌بستگی ۰/۶۰) و با خرده‌مقیاس کیفیت زندگی (ضریب هم‌بستگی ۰/۵۰) مشاهده شد. نتیجه‌گیری: نسخۀ فارسی IKDC ابزاری با پایایی و تکرارپذیری مطلوب در ارزیابی و طراحی برنامه‌هایپرسش‌نامۀتوان‌بخشی بیماران ایرانی، پس از جراحی رباط صلیبی قدامی‌ و منیسک زانوست.

Title: تأثیرحرکت‌درمانی با محدودیت اجباری به‌عنوان طرح مورد علاقۀ کودک بر کارکرد یک‌دستی و دودستی کودکان فلج مغزی نیمۀ بدن

http://rehabilitationj.uswr.ac.ir/article-1-985-fa.html

هدف: حرکت‌درمانی با محدودیت اجباری، روشی است که تأثیر آن بر افراد بزرگسال به اثبات رسیده است؛ اما درزمینۀ کودکان، هنوز نیاز به پژوهش‌های بیشتر احساس می‌شود. هدف از این مطالعه، بررسی تأثیرحرکت‌درمانی با محدودیت اجباری اصلاح‌شده، بر کارکرد دودستی و یک‌دستی کودکان با فلج نیمۀ بدن است. روش بررسی: این پژوهش با طرح مداخله‌ای، روی ۲۸ شرکت‌کننده ۱۰-۵ سال انجام شد که بنابر ملاک‌های ورود و کنارگذاری، در دو گروه قرار گرفتند: حرکت‌درمانی با محدودیت اجباری (میانگین سنی ۱٫۹۰±۹۴ ماه) و گروه کنترل (میانگین سنی۱۷٫۵۱±۸۵ ماه). مداخله در گروه حرکت‌درمانی با محدودیت اجباری به‌صورت ۶ ساعت در هرروز و طی ۱۰ روز متوالی در مراکز توان‌بخشی انجام شد که گروه کنترل از درمان رایج کاردرمانی در کلینیک استفاده می‌کردند. ابزارهای به‌کاررفته در این پژوهش، آزمون کفایت حرکتی بروینینکس‌ازرتسکی و آزمون کارکرد دستی جبسون‌تیلور و مقیاس بررسی استفادۀ کارکردی توسط مراقب بوده است. داده‌ها با آزمونهای تی زوجی و مستقل و کولموگروف-اسمیرنوف تحلیل شد. یافته‌ها: به‌منظور مقایسۀ دو گروه، از آزمون تی مستقل برای اختلاف نمرات استفاده شد و در متغیرهای چیره‌دستی (۰/۰۳۵=P)، هماهنگی دوطرفه (۰/۰۱۹=P)، هماهنگی اندام فوقانی (۰/۰۳۳=P)، فعالیت‌های دودستی (۰/۰۱۲=P)، فعالیت‌های یک‌‌دستی (۰/۰۲۱=P)، آزمون جبسون‌تیلور (۰/۰۳۵=P)، ادراک والدین (فراوانی) (۰/۰۰۵=P) و ادراک والدین (کیفیت) (۰/۰۰۱=P) تفاوت معنی‌داری بین دو گروه دیده شد. این متغیرها در سطح معنی‌داری (۰/۰۵=P) در گروه CIMT تفاوت نشان دادند؛ اما در گروه درمان رایج، تفاوت کمی‌دیده شده بود که معنی‌دار نبود. نتیجه‌گیری: روش حرکت‌درمانی با محدودیت اجباری موردِعلاقۀ کودک، بر کارکرد یک ‌‌دستی مؤثر بوده است و هم، با کمی‌تأمل، بر کارکرد دودستی کودکان با فلج نیمۀ بدن و استفاده از این پروتکل ممکن است در بهبود کارکرد دستی کودکان مؤثر باشد.
